# Human cancer germline antigen-specific cytotoxic T cell—what can we learn from patient

**DOI:** 10.1038/s41423-020-0468-x

**Published:** 2020-05-25

**Authors:** Megat Abd Hamid, Yanchun Peng, Tao Dong

**Affiliations:** 1 Nufield Department of Medicine, Chinese Academy of Medical Science Oxford Institute (COI), University of Oxford, Oxford, UK; 2 MRC Human Immunology Unit, MRC Weatherall Institute of Molecular Medicine, University of Oxford, Oxford, UK

**Keywords:** cytotoxic T cell, immune check point receptor, immune therapy, T cell receptor, cancer antigen, Cancer immunotherapy, Cellular immunity

## Abstract

In this review, we will highlight the importance of cancer germline antigen-specific cytotoxic CD8^+^ T lymphocytes (CTL) and the factors affecting antitumor CTL responses. In light of cancer immunotherapy, we will emphasis the need to further understand the features, characteristics, and actions of modulatory receptors of human cancer germline-specific CTLs, in order to determine the optimal conditions for antitumor CTL responses.

## Introduction

Cancer is one of the leading causes of mortality worldwide, with lung, colorectal, and liver cancer being the most common types of tumors.^1,2^ The small success rates in certain types of patients treated with current conventional cancer therapy such as radiotherapy and chemotherapy is contributed to not only by the multidrug resistance of cancer cells, but also by the low immunogenicity of tumors and the suppressive tumor microenvironment to immune cells.^3–5^ Therefore, cancer immunotherapy in which treatment is administered to patients to promote or restore the priming and antitumor activities of T cells has been of increasing interest. It is likely that combining immunotherapy with conventional treatment would allow better antitumor responsiveness and lead to cancer elimination.

Numerous clinical studies have established that the presence and enrichment of tumor-infiltrating T lymphocytes (TILs) in solid tumors is highly associated with better outcome in cancer patients.^6–10^ Importantly, tumors with enriched TILs (hot tumors) are highly receptive to immunotherapy compared with TILs-diminished tumors (cold tumors).^11,12^ In addition, studies using murine models further showed that adoptive transfer of T cells into tumor bearing mice exhibited significant tumor regression and improved overall response rate.^13–16^

Antitumor T-cell responses are critical to impeding cancer progression and can be targeted for therapy: clinical trials that block inhibitory receptors (IRs), such as PD-1 and CTLA-4, have shown great success. However, not all patients respond to the current immune-checkpoint inhibitors.^17–19^ In addition, the risk of causing autoimmune disease is high—largely due to the nonspecific nature of the approaches.^20–22^ New therapies that target cancer-specific immune cells are needed to improve patient outcomes, but our understanding of how cancer-specific T-cell responses go awry in patients is limited. To make progress, we must delineate the diverse mechanisms exploited by evolving tumors that cause T cells to lose their ability to detect and eliminate cancer cells in specific patients, and determine to what extent these functions can be restored. Furthermore, it is key to investigate how potential toxic effects may be controlled and T-cell exhaustion could be prevented.

The presence of T cells with impaired function correlates with poor patient prognosis in several types of cancers and chronic infections.^22^ Effective immunotherapies can augment cancer-specific immunity by improving the effector cell functions of cytotoxic T cells or the antigen presentation capacity of antigen presenting cells (APC) against cancer cells.

However, multiple factors will affect the quality of T-cell responses such as antigenic variation, the tumor microenvironment, and host genetics.^23–25^ Little is known in human settings about how these factors influence the net function of cancer-specific cytotoxic T lymphocytes (CTL), or the extent to which they synergize to silence cancer-specific CTLs in patients. A clearer understanding of the biologic and molecular aspects of CTL dysfunction in large patient cohorts is needed. We must not only develop more efficient strategies for checkpoint blockade, but also develop new therapies (combination or monotherapies) to deliver specific T-cell immunotherapy with optimal activation to prevent T-cell exhaustion or host pathology.

**Fig. 1:**
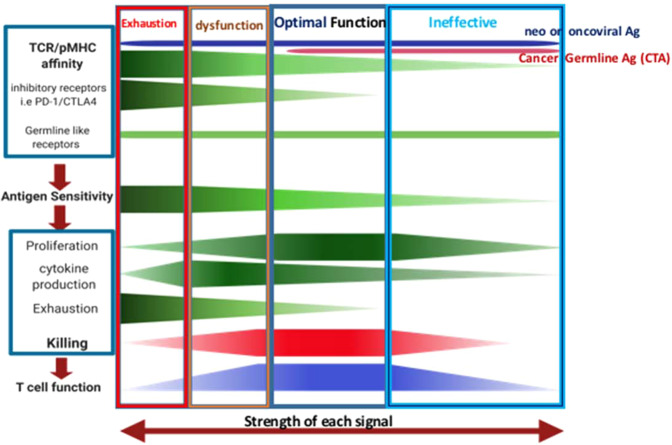
Optimal human cancer-specific T-cell responses are determined by multiple factors: (1) T cells express specific TCRs with weak affinity to MHC loaded with self-antigenic peptides including tumor associated (TAA) or cancer germline antigens (CTA), while neoantigen or viral-onco antigen (foreign) would span the full range of affinities, especially high affinity TCRs. When strong and consistent stimulation of a T cell occurs, it becomes exhausted and starts to express inhibitory receptors like PD-1 and CTLA-4. There is also a class of inhibitory as well as co-stimulatory receptors expressed on CTL, independent of antigen stimulation and cell activation, such as CD94/NKG2A and CD103. (2) Together these factors determine the overall antigen sensitivity of T cells, and (3) the level of subsequent T-cell functions such as: killing ability, proliferation, energy consumption, etc. (4) The optimal function of T cells is determined by immune balance between all these factors, such that a T cell may preserve its ability to kill its target cells, to produce cytokines, and to migrate whilst preventing exhaustion and death. The reason why cancer cells may grow in an uncontrolled fashion is likely due to T cells in their environment becoming dysfunctional/exhaustion or ineffective as a result of too low or too high antigen sensitivity. Combination of approaches to maintain T-cell optimal function and specificity is the key to controlling cancer development in patients

The current hypothesis is that optimal cancer-specific CTL function is determined by antigen sensitivity, which is influenced by T-cell receptor (TCR) affinity to different tumor antigens peptide loaded onto MHC class Ia molecules and/or nonclassical MHC class Ib molecules, such as HLA-E. In contrast to MHC class Ia, HLA-E are often highly upregulated in tumors. Furthermore, cancer-specific CTL function is modulated by IRs and co-stimulatory receptors that accumulate during disease progression. Disturbance of the fine balance of these factors alters optimal T-cell function and contributes to cancer development and metastases (Fig. 1).

## Tumor antigen-TCR recognition and its usage in cancer immunotherapy

### Cancer germline (testis) antigens (CTA)

Among the different classifications of cancer antigens, the primary classes that are at present widely used in cancer immunotherapy research are cancer neoantigens, oncoviral antigens, and CTA. Neoantigens are a class of antigens that is completely foreign and absent in normal human tissues and cells while present exclusively on cancer cells. In contrast, oncoviral antigens are peptides expressed by virally associated tumors, such as oncoprotein E7 of human papillomavirus 16.^26,27^ Neoantigen-specific and oncoviral antigen-specific T cells bearing TCRs of full range of affinities to pMHC, especially high affinity T cells. Following chronic stimulation of neoantigen-/oncoviral antigen-specific T cells, they can become exhausted and dysregulated, with expression of IRs such as PD-1 and CTLA-4.^28,29^

CTAs are a class of antigens not typically expressed by normal cells outside of the testes—cells of which do not express MHC class I, nor present antigens to CD8^+^ T cells. However, CTAs are frequently expressed on cancer cells.^30^ Human CTAs, such as synovial sarcoma X-2 (SSX-2), New York-esophageal squamous cell carcinoma-1 (NY-ESO-1), and melanoma associated antigen A-1 or A-3 are over-expressed in different human cancers such as in melanoma and lung cancer.^31–33^ CTA-specific T cells are frequently detected in cancer patients and contribute to tumor regression. Several studies using CTA vaccine strategy have demonstrated correlation between increased CTA-specific T-cell responses with progression-free survival.^34,35^ This benefit is further confirmed by another study showing that CTA^+^ patients receiving adoptive transfer of autologous T cells transduced with NY-ESO-1-specific TCR exhibited better clinical responses.^36^ However, due to the nature of CTA antigens, TCRs used by T cells specific to these antigens possibly have lower affinity for pMHC and exhibit weaker antigen sensitivity or lower functional avidity. Although CTA-specific T cells are less likely to express high level of activation-induced IRs,^37–39^ some of them can demonstrate upregulation of germline-like IR expression, such as CD94/NKG2a with compromised antitumor activity.^40^ Exploring how to improve antitumor activities of CTA-specific CTLs could provide profound and broader clinical benefits to cancer patients.

### Engineered TCR-T cells strategy for cancer immunotherapy

Engineered cancer-specific TCR-T cells is one of the current immunotherapy strategies designed with the aim of improving antitumor T-cell responses in patients. It is a directed therapy using TCR of T cells that specifically target/recognize cells expressing particular antigen such as CTAs. Careful selection of TCRs that selectively recognize cancer pMHC molecules on tumors but not on normal tissues—form a vital aspect of this approach.^41,42^

In humans, the TCR diversity is estimated to be up to 10^18^ variations, due to the random DNA rearrangement of each of the TCR chain and in the random pairing of TCR α and β chains (as well as γδ TCRs) on individual T cell.^43–45^ Each TCR recognizes specific antigen, such as viral, bacterial, or oncogenic antigens bounded on specific MHC type (with diverse MHC alleles present). Considering the large variety in TCR repertoire and antigen specificities, the canonical method of selecting the correct TCRs for engineering is through sequencing TCR α chain and β chain mRNAs from in vitro-cultured T cells of cancer patients, or using algorithmic predictions based of the CDR3 binding region of TCRs.^42,46–48^ The highly specific and directed nature of TCR–pMHC interaction prevent engineered TCR-T cell from targeting cells that do not express the specific tumor antigen and MHC restriction.

Significant success has been highlighted in various preclinical and clinical studies in human with different types of cancer including synovial, myeloma, colorectal carcinoma, and melanoma. A study in 2006 using engineered MART-1-specific TCR-T cells demonstrated significant tumor regression in treated melanoma patients.^49^ Another study by Rosenberg et al. has shown that more than 19% of metastatic melanoma patients receiving adoptive transfer of gp100-specific TCR-T cells exhibited strong antitumor T-cell responses posttreatment.^50^ Various other studies have gone into clinical trials including TCR-T cells that target CTA pMHC molecules. Treatment using TCR-T cells targeting HLA-A0201-restricted NY-ESO-1 cancer antigen exhibited improved overall clinical response rate in more than 50% of melanoma, synovial or multiple myeloma patients, with no off-site toxicities observed.^36,51,52^ Importantly, prolonged presence of TCR-T cells was detected in a clinical trial using HLA-A2-restricted MAGE-A4-specific TCR-T cells in treated esophageal cancer patients, with three patients having minimal lesions at 27 months posttreatment.^53^ These clinical successes highlighted the importance and potential benefits of using precise and directed cancer immunotherapy strategy specifically targeting tumor antigens-expressing cancer cells.

## Cancer immune evasion and the impacts on CTL responses

However, cancers are now well-established to employ diverse mechanisms to escape antitumor immunity. Among the known escape mechanisms include having reduced TCR/pMHC affinity, downregulation of the pMHC class Ia surface expression, chronic antigen exposure, and elevated expression of IRs such as PD-1. Particularly for CTLs, it can result in having impaired activation and proliferation, decreased production of inflammatory cytokines, and limited or inefficient cancer cell killing. These factors contribute to anergic and exhaustive state of T cells in tumor, preventing CTLs from performing optimally in their antitumor functions (Fig. 1).

### Downregulation of MHC class Ia leads to compromised T-cell priming, activation, and ineffective responses

One of the first known hallmarks of cancer cells is their ability to downregulate MHC class Ia expression on their cell surface. This observation is well-established in multiple cancer types, including in melanoma and lung cancers.^54,55^ The downregulation of MHC class Ia expression is thought to involve the dysregulation of antigen processing machinery such as transporter-associated protein (TAP), tapasin, and calnexin. Resultant inefficient antigen processing leads to reduced presentation of stabilized pMHC at the cancer cell surface.^55^ In turn, the lack of pMHC presentation can impair cancer-specific CTLs’ ability to identify and kill cancer cells, as the pMHC downregulation masks the dysregularity and presence of oncogenic cells. Interestingly, our group has recently shown that this downregulation was not only observed on tumor cells, but also on the APCs in the tumor microenvironment such as on the tumor-residing CD141^+^ conventional dendritic cells (cDC) and macrophages.^40^ As APCs are critical for mediating adaptive immune responses, the overall downregulation of pMHC in the tumor microenvironment may lead to a compromised T-cell priming and activation.

### Exhausted T cells exhibit elevated inhibitory receptor expression

It is now well-established that chronic tumor antigen stimulation and the immunosuppressive tumor microenvironment have driven T cells to exhaustion. Exhausted TILs exhibit upregulated expression of multiple IRs such as PD-1, CTLA-4, and Tim3. The immunosuppressive tumor microenvironment could present the necessary ligands to these IRs which, in turn, impairs, limits, and exhausts antitumor T-cell activities. Due to their elevated presence in cancer, the primary focus of current immunotherapy has been on immune-checkpoint blockade treatment, in order to block the inhibitory effects of IRs so as to allow T cells to recover their antitumor responses.

CTLA-4 was the first IR found to negatively affect TILs’ functions. In healthy condition, CTLA-4 is an IR inducible upon T-cell activation. CTLA-4 recognizes B7-1 (CD80) and B7-2 (CD86), the same ligands that can be recognized by the co-stimulatory receptor CD28. In contrast to CD28, CTLA-4 negatively regulates the activation of T cells.^56^ In preclinical cancer models, CTLA-4 blocking antibody treatment in mice was found to successfully prevent tumor establishment and also promote tumor rejection.^57,58^ However, in human clinical studies, fully humanized anti-CTLA-4 treatment, such as ipilimumab, was shown to increase the risk of autoimmune diseases such as colitis in certain treated cancer patients.^59,60^

PD-1 is well-established to contribute to T-cell exhaustion in cancer. PD-1 interact with its ligands, PD-L1 or PD-L2, and can mediate the epigenetic transformation of T cells, creating hyporesponsive a T-cell phenotype that resembles senescence, leading to cell cycle arrest and reduced cytolytic activities of CTLs.^61–63^ Activation of PD-1 on T cells phosphorylates the downstream inhibitory molecule SHP-1, a protein known to negatively affect the cascade signaling of TCR by interfering with the phosphorylation of ZAP70 and PKCθ, impairing—among other factors—the calcium utilization of T cells.^64,65^

In tumors, both PD-1 and PD-L1 are known to be upregulated on TILs and cancer cells. Preclinical studies blocking the PD-1/PD-L1 axis demonstrated increased overall T-cell activation, antitumor T-cell responses, and inhibition of murine tumor growth.^66,67^ In humans, fully humanized anti-PD-1 antibodies such as pembrolizumab and anti-PD-L1 antibodies such as atezolizumab have demonstrated significant tumor regression, such as in melanoma, lung and renal carcinoma patients.^68–70^ Furthermore, it was found that metastatic patients with high PD-L1 expression treated with pembrolizumab exhibited active proliferation of cytotoxic TILs, with decreasing tumor size.^11^ It is therefore not surprising that pembrolizumab was successfully certified by the US FDA in 2014 for the treatment of metastatic and late stage cancer patients. However, it is worth noting that treatment with anti-PD-1 or anti-PD-L1 failed to improve outcome in certain cancer patients and cancer types. It is therefore likely that other mechanisms could be at play, either by compensating loss of the PD-1/PD-L1 axis or by imposing inhibitory effects on TILs independently from influence of PD-1/PD-L1.

### Other known inhibitory receptors

Other IRs have also been discussed as potential targets for cancer immunotherapy, including Tim3, BTLA, and TIGIT. Tim3 was originally found to suppress the effector Th1 responses in murine model of multiple sclerosis.^71^ In chronic viral infection, Tim3 expression is highly persistent and is associated with the inflammatory regulation of T cells.^72^ In cancer, Tim3^+^ TILs in lung cancer patients exhibited significant defects in cytokine production and T-cell proliferation.^3,73^ Furthermore, co-expressed Tim3^+^ PD-1^+^ TILs were demonstrated to represent a deeply exhausted T-cell population, with a strong association to tumor progression such as in prostate, colorectal, and hepatocellular carcinoma.^74,75^

In contrast, TIGIT was found to inhibit spontaneous T-cell activation, and is primarily expressed following T-cell activation and on memory T cells.^76^ BTLA is also expressed following T-cell activation and phosphorylation of this receptor is known to interfere with the downstream TCR-mediated signaling cascade.^77^

A recent study in our group has shown that the expression of BTLA, TIGIT, KLRG-1, and 2B4 is not enriched on TILs compared with their counterparts in peripheral blood of cancer patients.^78^ Most importantly, we found that co-expression of specific IRs including Tim3 and PD-1 is highly prevalent on CD8^+^ TILs across different cancer types, such as in lung, kidney, and breast cancers. This suggests that certain IRs are likely more favored in the tumor microenvironment to suppress antitumor CTLs responses. The co-expression of multiple IRs on TILs indicates the possibility that mono-immunotherapy targeting a single IR might not be enough, as there could be compensatory inhibitory mechanisms taking place. Therefore, as others in the field have suggested, a combinatory immune-checkpoint blockade immunotherapy strategy could provide better clinical responses in cancer patients.

## Co-stimulatory receptors and their importance in antitumor CTLs responses

In contrast to IRs, co-stimulatory receptors of T cells act as a secondary signal for an effective TCR-mediated T-cell activation and immune response. Current well-established co-stimulatory receptors of T cells include CD28, 4-1BB, and ICOS. It is known that an overall activation of T cells without co-signaling can lead to T-cell anergy, a state of hypo-responsiveness and growth arrest, and contribute toward dysregulated immune tolerance and impaired effector functions.^79^

CD28 is the best characterized co-stimulatory receptor of activated T cells, acquired only following activation. Its ligation to CD80 or CD86 promotes T-cell proliferation, cytokine production, cell survival, and stabilization of cytokine mRNAs.^80–82^ Studies using a CD28-deficient murine model highlighted the necessity of CD28. In this study, its absence was shown to result in an ineffective effector response and a lack of viral control.^83^ In addition, adoptive transfer of CD80-transfectant tumor cells into mice produced a strong CD28-responsive CTL-mediated immune responses and tumor rejection.^84^ As CD28 and CTLA-4 share the same ligands of CD80 and CD86,^56^ these two receptors heavily compete for the same ligand recognition and therefore can affect and define the overall activatory or inhibitory outcome of T-cell activities.

4-1BB: similarly to CD28, both 4-1BB and ICOS are only expressed by T cells following T-cell activation. For 4-1BB, its ligation to 4-1BBL, expressed mainly on dendritic cells, allows conducive effector CTL responses.^85,86^ A preclinical study using 4-1BB agonist in combination with anti-PD-1 treatment in a tumor bearing murine model demonstrated an elevated IFNγ response, mediated primarily by the effector memory CTLs.^87^ In a human setting, a phase I clinical trial with 4-1BB agonist (utomilumab) showed not only its safety, but also that the agonistic antibody induced higher overall response rates in melanoma patients.^88^ However, <4% of nonmelanoma cancer patients with solid tumors treated with this regiment exhibited improved responses. Another anti-4-1BB antibody (urelumab) used in clinical trial for treatment of advanced cancers unfortunately induced severe liver toxicity in some of the patients treated, likely due to nonspecific immune cells activation that may cause tissue damage.^89^

ICOS, also known as CD278, is present on activated T cells and interacts with its ligand, ICOS-L, expressed mainly on APCs. Activation of ICOS was first known to enhanced T-cell proliferation.^90,91^ Interestingly, preclinical studies demonstrated that ICOS was upregulated following anti-CTLA-4 treatment in tumor bearing murine models, suggesting that it could be an important mediator of better T-cell responses in the anti-CTLA-4 treated patients.^92,93^

## Germline-like receptors and their potential role in optimal CTL responses

Progression of CTLs into an exhausted or dysfunctional state is mainly caused by chronic antigen exposure, inefficient T-cell activation and the upregulation of activation-associated IR expression, such as PD-1. The immunosuppressive tumor microenvironment nudges TILs into an exhausted state, and prevents optimal antitumor T-cell responses (Fig. 1). Constitutive markers of T cells include germline-like receptors such as CD103 and NKG2a, which, with their ligands continually presented robustly on most cancer cells, are much less studied for their function as co-stimulatory or co-IR for CTLs. These germline-like receptors could be critical to understanding the optimal conditions for effective antitumor T-cell immune responses.

### CD103, more than a tissue residency marker

CD103, also known as integrin alpha E, was first discovered as a T-cell marker for tissue retention and residency, especially in the gut and lung. It forms heterodimeric pairing with integrin beta 7 to form a complete complex recognized by E-cadherin, a molecule expressed at moderate level primarily by epithelial cells. Interaction between CD103 and E-cadherin allows attachment of CD103^+^ immune cells to tissue.^94,95^ Also termed as CD103^+^ tissue-resident memory T cells (CD103^+^ Trm cells), they lack tissue exit markers such as CD62L, CCR7, S1PR1, and CX3CR1, while upregulating Notch transcription factor and pro-survival molecules such as Bcl-2 and BIRC3.^96–102^

**Fig. 2:**
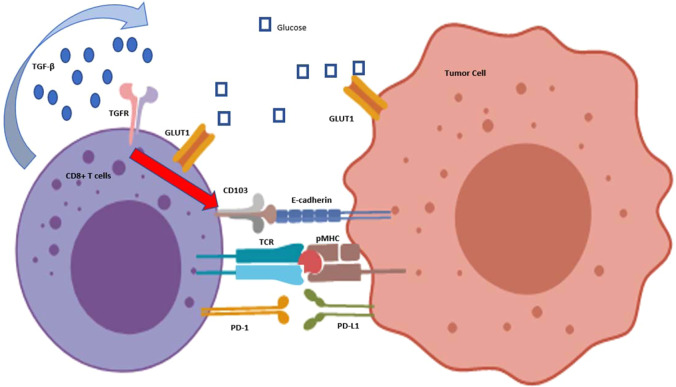
Characteristics of CD103 +  CTA-specific CTLs. Patient circulation-derived CTA-specific CD103+ CTLs exhibit self-production of TGF-β1 in its active form, which is potentially recognized by self-TGFR, mediating the self-maintenance of CD103 expression. This unique T-cell population also has elevated glucose consumption over time, and mediates faster recognition and killing of individual cancer cells. The presence and interaction between CD103 and its ligand E-cadherin on cancer cells would support the faster recognition. However, the presence or absence of PD-1 and its interaction with PD-L1 on cancer cells might influence the antitumor T-cell responses

CD103 expression is known to be acquired during naive T-cell development, following initial antigen pMHC contact and in the presence of TGF-β1.^103,104^ Notably, Batf3-dependent murine DC and human CD1c^+^ cDC were defined as the primary producer of TGF-β1 and antigenic contact during the development process.^105–107^ Recently, our group has surprisingly found the presence of matured CD103^+^ CTA-specific CTLs from the peripheral blood of cancer patients.^108^ Most importantly, this unique T-cell population can self-produce activated TGF-β1 through its own membrane β8 expression to sustain its CD103 expression (Fig. 2).

CD103^+^ T cells were found to help mediate better immune surveillance. For example, enrichment of Trm cells in tissue resulted in faster clearance of influenza murine lung infection and HSV murine skin infection.^96,109^ In cancer, murine studies have shown that the presence of CD103 on TILs contributes to elevated T-cell infiltration into solid tumors, with better tumor control.^110,111^ Consistent with its known role as a retention marker, our group and others have shown that CD103^+^ TILs can home and localize better to E-cadherin^+^ tumor islets.^97,108,111^ Importantly, we found that they clustered at a higher density within and surrounding the E-cadherin^+^ tumor islets, compared with the CD103^−^ TILs^108^ (Fig. 2). In addition, epithelial-derived cancer cells are also known to over-express E-cadherin on their surface. Altogether, it is therefore likely that CD103^+^ TILs could play a significant role in inducing better antitumor immune responses.

Interestingly, studies by Mami-Chouaib’s group have demonstrated an accumulation of CD103 molecules within the point of contact between CD103^+^ T cells and autologous E-cadherin^+^ cancer cells.^112,113^ The lack of E-cadherin on cancer cells can also diminish the movement of CD103 into the interface. Surprisingly, they showed that CD103 accumulation does overlap with an enriched granzyme B presence within the synapse. This suggests the possibility that CD103 could provide additional co-stimulatory signaling for antitumor CTL cytotoxic responses, upon its interaction with E-cadherin. This notion is supported by other studies associating the enrichment of CD103^+^ TILs with better clinical outcome in patients with either melanoma, colorectal or lung cancer.^100,114,115^ Also, preclinical studies found that the lack of E-cadherin on some melanoma cells can impair the overall antitumor responsiveness of murine CD103^+^ Trm cells, even after anti-CTLA-4 and anti-PD-1 treatments.^116^ However, it was unclear how exactly the presence of CD103 contributes toward tumor control.

As mentioned previously, TCR-CTA pMHC recognition is of low affinity, and this can reduce the overall activation and response of CTA-specific CTLs. Using circulation-derived, TCR-matched CD103^+^ and CD103^−^ CTA-specific CTLs, our group recently showed that the presence of CD103 can help to significantly elevate TCR antigen sensitivity.^108^ In particular, we found that TCR-matched CD103^+^ CTA-specific CTLs produce and secrete elevated inflammatory cytokines such as IFNγ compared with the TCR-matched CD103^−^ CTLs. Most importantly, this unique T-cell population exhibits faster recognition of individual cancer cells and induces rapid antitumor cytotoxicity, primarily through degranulation-mediated cytotoxicity. This form of cytotoxicity is supported by a previous study showing elevated granzyme B accumulation in the CD103^+^ T-cell immunological synapse.^112,113^ Using live cell imaging, we further demonstrated that CD103^+^ CTA-specific CTLs induce faster E-cadherin^+^ cancer cell killing compared with the TCR-matched CD103^−^ CTLs.^108^ This rapid killing allowed significantly higher numbers of accumulative E-cadherin^+^ cancer cell deaths over time. The elevated antigen sensitivity, however, was impaired following either treatment with anti-CD103, or in the absence of E-cadherin on cancer cells. It is therefore very likely that the presence of CD103 on CTA-specific CTLs and its engagement with E-cadherin on cancer cells help to either improve the rapidness of immunological synapse formation, strengthening the interaction kinetics of TCR–pMHC binding and/or activate downstream signaling that acts as a co-signal to the TCR-mediated activatory signaling.

An efficient antitumor response requires high energy usage, particularly in producing and trafficking cytokines and cytolytic molecules into the immunological synapse via the action of cytoskeletal activities.^117,118^ It is therefore not surprising that our group found that the CD103^+^ CTA-specific CTLs have elevated glucose consumption over time, with higher maximal capacity to metabolize glucose compared with the TCR-matched CD103^−^ CTLs.^108^

However, in the tumor microenvironment, enhanced glucose consumption by cancer cells can deprive TILs of the essential glucose needed for proper immune functioning.^119^ Interestingly, a recent study by the Kupper group has demonstrated that virus-specific CD103^+^ Trm cells are highly capable of utilizing exogenous free fatty acids via their mitochondria, and this contributed toward enforcing protective immunity against virus-infected tissue.^120^ Importantly, our group has further shown in cancer settings that the CD103^+^ CTA-specific CTLs have elevated overall mitochondrial activities and maximal mitochondrial spare capacity.^108^ This therefore suggests that in the case of glucose-deprived microenvironments such as in cancer, CD103^+^ CTA-specific CTLs could compensate the lack of glucose metabolism with other non-glucose metabolisms to meet their high energy demand (Fig. 2).

### Upregulated CD94/NKG2a and its ligand HLA-E in cancer

Another germline-like receptor that we found highly expressed in cancer is the heterodimeric CD94/NKG2a.^40^ This receptor was originally found on NK cells, with its first known function being to limit NK cell cytotoxicity and prevent autoimmunity in healthy tissue.^121–125^

Our group was able to establish CTA-specific CTL clones with matched TCR, but different surface expression, such as with either CD103 or CD94/NKG2a.^40,108^ Using the NKG2a^+^ and NKG2a^−^ TCR-matched CTA-specific CTLs in a recent study, we found that exposure of CD94/NKG2a^+^ CTLs to HLA-E-enriched cancer cells significantly impaired the degranulation-mediated cytotoxicity and the production and secretion of inflammatory cytokines and chemokines such as IFNγ and CCL-5.^40^ This was further demonstrated in an ex vivo approach, using freshly isolated CD94/NKG2a^+^ TILs from HLA-E-enriched tumor tissue. In addition to its effect on killing, the enriched HLA-E and CD94/NKG2a interaction significantly affected IL-2-dependent T-cell proliferation, in which the production and secretion of IL-2 were detrimentally impaired and the expression of IL-2 receptor alpha (CD25) was also reduced. Similarly to previous studies, however, the use of anti-NKG2a can reverse the negative effect of NKG2a on antigen-specific CTL activities.^40,126,127^

This mechanism is mediated through CD94/NKG2a interaction with the nonclassical MHC class Ib molecule, HLA-E on normal cells. In contrast to the MHC class Ia, numerous studies have now found that HLA-E as well as HLA-G are enriched in various types of cancer.^128–131^ Tumors with either upregulated HLA-E or dually upregulated HLA-E and HLA-G are associated with poorer clinical outcome in patients.^129^ Importantly, our group recently demonstrated a significant correlation between HLA-E-enriched tumors with CD94/NKG2a-enriched TILs, in four different cancer types.^40^ This suggest that CD94/NKG2a and HLA-E could negatively affect antitumor TILs responses. A recent phase II clinical trial using anti-NKG2a and anti-EGFR to treat head-and-neck cancer patients showed an improved overall response rate in the majority of patients treated, further highlighting the potential of HLA-E and NKG2a as cancer immunotherapy target combination.^126^ However, it was unclear the mechanisms by which HLA-E and CD94/NKG2a could interfere with the antitumor CTL responses.

Overall, these examples of germline-like receptors such as NKG2a and CD103 highlight the significance of identifying and exploring germline-like receptors, particularly of cancer-specific T cells.

## Cytokine defect and the implication on identification of cancer-specific CTLs in patients

Alongside directed cytotoxicity, CTLs function as to produce important inflammatory cytokines such as IFNγ and TNFα. IFNγ was first discovered to interfere with viral replication in host, and the activation of IFNγ receptor-mediated JAK1/STAT-1 signaling enables the formation of immunoproteasome and the expression of antigen processing machinery-associated genes such as TAP and tapasin on target cells.^132–135^ In contrast, TNFα ligation to TNF receptors 1 and 2 promotes receptor-ligand-mediated apoptosis events and prevents the expression of antiapoptotic factors such as Bcl-2.^136,137^

In cancer, preclinical studies using IFNγ-deficient tumor bearing murine model demonstrated reduced migration of activated T cells into the tumor sites, with enhanced tumor progression.^138,139^ Surprisingly, a recent study in our group found a sizeable numbers of SSX-2-specific CTL clones from a cancer patient that lack any IFNγ mRNA and protein expression.^140^ These IFNγ^−^ CTL clones, however, have normal production of TNFα and other cytokines. In addition to these clones, a sizeable portion of short-term expanded NY-ESO-1-specific and SSX-2-specific CTL lines from two other cancer patients exhibited hypermethylation of their IFNγ gene promoter. DNA hypermethylation prevents the recruitment of essential DNA transcription machinery proteins such as DNA polymerase onto the gene, which can limit the level of specific DNA transcription and protein synthesis in a cell.^141,142^ The inaccessibility to the IFNγ gene promoter can therefore result in a dysregulated cytokine production and contribute to an ineffective antitumor CTA-specific CTL responses. In addition to IFNγ, other cytokines deficiency in tandem may also be exhibited and be detrimental to the antitumor responses of CTA-specific CTLs.

Current methods to identify cancer-specific CTLs rely on the IFNγ expression and production following stimulation, such as by using intracellular cytokine assay and enzyme-linked immunosorbent assay.^139,143,144^ Our group has recently demonstrated a certain portion of CTA-specific CTLs could be deficient in their IFNγ expression.^140^ This suggest that the commonly used techniques might have overlooked a great proportion of CTA-specific CTLs and could potentially lead to unintended biases in cancer-specific T cells analysis. It is therefore important that more diverse methods that can detect multiple cytokines be considered for a proper identification and analysis of CTA-specific CTLs from cancer patients.

## Concluding remarks

Optimal cancer-specific CTL function is determined by antigen sensitivity, which is influenced by TCR affinity to different tumor antigens peptide loaded onto MHC class Ia molecules and/or nonclassical MHC molecules such as HLA-E. In contrast to MHC class Ia molecules, HLA-E are highly upregulated on cancer cells and APCs in the cancer microenvironment. Furthermore, cancer-specific CTL function is modulated by IRs and co-stimulatory receptors that accumulate during disease progression. Disturbance of the fine balance of these factors alters optimal T-cell function and contributes to cancer development and metastasis. Understanding the quality and key features of human cancer germline antigen-specific T cells in cancer patients and applying this knowledge to immune therapy should be one of the key priorities to consider. In comparison to exhausted T cells—which are likely unrestorable—this approach may help to restore dysfunctional as well as push ineffective immune cells to their optimal condition—through manipulating TCR or co-receptors of T cells (Fig. 1).
